# Adjuvant TACE may not improve recurrence-free or overall survival in HCC patients with low risk of recurrence after hepatectomy

**DOI:** 10.3389/fonc.2023.1104492

**Published:** 2023-05-24

**Authors:** Long-Hai Feng, Yu-Yao Zhu, Jia-Min Zhou, Miao Wang, Wei-Qi Xu, Ti Zhang, An-Rong Mao, Wen-Ming Cong, Hui Dong, Lu Wang

**Affiliations:** ^1^ Department of Hepatic Surgery, Shanghai Cancer Center, Fudan University, Shanghai, China; ^2^ Department of Oncology, Shanghai Medical College, Fudan University, Shanghai, China; ^3^ Department of Pathology, Eastern Hepatobiliary Surgery Hospital, The Second Military Medical University, Shanghai, China

**Keywords:** hepatocellular carcinoma, hepatectomy, postoperative adjuvant TACE, low risk of recurrence, prognosis

## Abstract

**Background:**

To identify whether adjuvant transarterial chemoembolization (TACE) can improve prognosis in HCC patients with a low risk of recurrence (tumor size ≤ 5 cm, single nodule, no satellites, and no microvascular or macrovascular invasions) after hepatectomy.

**Methods:**

The data of 489 HCC patients with a low risk of recurrence after hepatectomy from Shanghai Cancer Center (SHCC) and Eastern Hepatobiliary Surgery Hospital (EHBH) were retrospectively reviewed. Recurrence-free survival (RFS) and overall survival (OS) were analyzed with Kaplan-Meier curves and Cox proportional hazards regression models. The effects of selection bias and confounding factors were balanced using propensity score matching (PSM).

**Results:**

In the SHCC cohort, 40 patients (19.9%, 40/201) received adjuvant TACE, and in the EHBH cohort, 113 patients (46.2%, 133/288) received adjuvant TACE. Compared to the patients without adjuvant TACE after hepatectomy, patients receiving adjuvant TACE had significantly shorter RFS (P=0.022; P=0.014) in both cohorts before PSM. However, no significant difference existed in OS (P=0.568; P=0.082). Multivariate analysis revealed that serum alkaline phosphatase and adjuvant TACE were independent prognostic factors for recurrence in both cohorts. Furthermore, significant differences existed in tumor size between the adjuvant TACE and non-adjuvant TACE groups in the SHCC cohort. There were differences in transfusion, Barcelona Clinic Liver Cancer stage and tumor-node-metastasis stage in the EHBH cohort. These factors were balanced by PSM. After PSM, patients with adjuvant TACE after hepatectomy still had significantly shorter RFS than those without (P=0.035; P=0.035) in both cohorts, but there was no difference in OS (P=0.638; P=0.159). Adjuvant TACE was the only independent prognostic factor for recurrence in multivariate analysis, with hazard ratios of 1.95 and 1.57.

**Conclusions:**

Adjuvant TACE may not improve long-term survival and might promote postoperative recurrence in HCC patients with a low risk of recurrence after hepatectomy.

## Introduction

Recurrence after resection is the main obstacle for hepatocellular carcinoma (HCC) patients and limits surgical efficacy (1, 2). Many adjunctive therapies have been used to reduce the risk of recurrence and metastasis after liver resection (3–5).

Transarterial chemoembolization (TACE) has been the most widely used treatment for intermediate-stage HCC and preoperative downstaging treatment (2, 6–8). Preventively, adjuvant TACE has been used to reduce the risk of recurrence and prolong survival for HCC patients after hepatectomy (9). And it is usually performed approximately 4 weeks after hepatectomy.

However, it remains controversial whether adjuvant TACE can benefit HCC patients after hepatectomy (10, 11). A growing amount of supporting evidence has confirmed that, for the high-risk recurrence population (tumor size > 5 cm, multiple nodules, circulating tumor cells, microvascular invasion (MVI) and macrovascular invasion), adjuvant TACE can significantly reduce the recurrence rate and prolong long-term survival (5, 12–18). The procedure might improve the prognosis by eliminating residual cancer cells. Interestingly, for HCC patients with a low risk of recurrence (tumor size ≤ 5 cm, single nodule, MVI-negative and no macrovascular invasions), it is unclear whether adjuvant TACE could provide benefits after hepatectomy.

Here, focusing on HCC patients with a low risk of recurrence, we reassessed whether adjuvant TACE could benefit prognosis in these patients after hepatectomy with a propensity score matching (PSM) analysis from two independent cancer centers.

## Materials and methods

Patients diagnosed with HCC who underwent hepatectomy between March 2015 and September 2019 at Shanghai Cancer Center (SHCC) in Shanghai, China, and patients diagnosed between December 2009 and June 2010 who underwent hepatectomy at the Eastern Hepatobiliary Surgery Hospital (EHBH) in Shanghai, China, were retrospectively reviewed.

The inclusion criteria were as follows: (1) HCC was diagnosed pathologically after hepatectomy; (2) single nodule; (3) diameter ≤ 5 cm; (4) Child–Pugh A or B liver function; and (5) adjuvant TACE adopted within 2 months after hepatectomy. The exclusion criteria were as follows: (1) recurrent HCC; (2) patients with microvascular or macrovascular invasions or satellites; (3) patients with extrahepatic metastasis; (4) a previous history of treatment of malignancy; (5) recurrence before or at adjuvant TACE; (6) perioperative mortality; and (6) incomplete data.

Data were extracted from medical records, cross-checked and statistically analyzed. The following clinicopathological parameters were extracted and analyzed statistically: sex, age, chronic hepatitis B/C virus (HBV/HCV) infection, transfusion, albumin (ALB), total bilirubin (TBIL), alanine transaminase (ALT), aspartate aminotransferase (AST), γ-glutamyl transpeptidase (GGT), alkaline phosphatase (ALP), alpha-fetoprotein (AFP), carbohydrate antigen 19-9 (CA19-9), prothrombin time (PT), platelet count, tumor size, differentiation of tumor cells and liver cirrhosis.

The chemotherapeutic regimens used in adjuvant TACE included hydroxycamptothecin, pirarubicin (THP) and floxuridine (FUDR) in the SHCC cohorts and doxorubicin hydrochloride, TPH and epirubicin in the EHBH cohorts. The dosages of these chemotherapeutic drugs and lipiodol were determined by body surface area and liver function.

This study was approved by the Clinical Research Ethics Committee of EHBH and the Institutional Review Board and the Ethics Committee of SHCC. Written informed consent was obtained from all subjects before the operation and (or) TACE. The consent was also obtained for participation in our study.

### Follow-up

Patients were followed up as in our previous report (19, 20). Recurrence and overall survival (OS) were used as endpoints. Recurrence-free survival (RFS) was calculated from the date of operation to the date when recurrence or metastasis was diagnosed. OS duration was defined as the interval between surgery and the time of death due to any cause. The most recent EHBH patient has been followed up for five years. The deadline for follow-up in SHCC patients was 03-31-2022. During the follow-up period, patients with recurrence or metastasis were treated with optimal therapeutic methods.

### Statistical analysis

MedCalc statistical software (version 19.3, Ostend, West-Vlaanderen, Belgium) was used to analyze the data acquired from this study (21). Continuous variables were analyzed with Student’s t test or the Mann–Whitney U test, and categorical variables were compared with the chi-squared test, Fisher’s exact test or Wilcoxon’s signed-rank test, where appropriate. Kaplan–Meier curves, log-rank tests and Cox proportional hazards regression analysis were used to analyze recurrence and survival. The effects of selection bias and confounding factors were balanced using PSM (nearest neighbor matching) with the *MatchIt* package in R software (version 4.1.2) (22). All statistical tests were two-tailed, and the difference was considered statistically significant when the *P* value was less than 0.05.

## Results

### Clinicopathological features of the adjuvant TACE and non-adjuvant TACE cohorts

Overall, 201 HCC patients from SHCC and 288 patients from EHBH were included in our study (**Supplementary Figure 1**). In the SHCC cohorts, 40 (19.9%, 40/201) patients received TACE after hepatectomy. Correspondingly, 113 (46.2%, 133/288) patients received it in the EHBH cohorts. Except for tumor size, there were no significant differences in clinicopathological parameters between adjuvant TACE and non-adjuvant TACE cohorts in the SHCC cohorts (**Table 1**). And, there were differences in transfusion, Barcelona Clinic Liver Cancer (BCLC) stage and tumor-node-metastasis (TNM, American Joint Committee on Cancer (AJCC), 8th) stage between adjuvant TACE and non-adjuvant TACE cohorts in the EHBH cohorts (**Table 2**). These factors were balanced by PSM with nearest neighbor matching. After PSM with 1:2 ratio matching in the SHCC cohort and 1:1 ratio matching in the EHBH cohort, the clinicopathological differences were well balanced between the adjuvant TACE and non-adjuvant TACE groups in both the SHCC and EHBH cohorts (**Tables 1**, **2**). Histograms of propensity scores before and after matching in the two cohorts are shown in **Supplementary Figures 2**, **3**.

**Table 1:** Clinicopathological features of hepatocellular carcinoma patients with low risk of recurrence after liver resection (Shanghai Cancer Center Cohorts).

Clinicopathological features	Adjuvant TACE (n=40)	Non-adjuvant TACE before a PSM (n=161)	Non-adjuvant TACE after a PSM 1:2 (n=80)	*P_1_* values	*P_2_* values
Sex, female/male	8/32 (20.0%/80.0%)	20/141 (12.4%/87.6%)	8/72 (10.0%/90.0%)	0.215	0.129
Age, range (years)	55.7±10.8 (35.0-81.0)	56.8±11.0 (31.0-84.0)	57.1±11.4 (31.0-84.0)	0.567	0.524
Hepatitis, Yes/No	29/11 (72.5%/27.5%)	126/35 (78.3%/21.7%)	60/20 (75.0%/25.0%)	0.438	0.768
TBIL, (μmol/L)	12.5 (8.9-18.3)	11.7 (9.1-15.8)	11.3 (8.6-15.1)	0.245	0.126
ALB, range (g/L)	43.4±3.9 (33.3-50.9)	44.2±3.5 (35.6-53.8)	43.7±3.5 (35.6-52.2)	0.194	0.605
ALT, range (U/L)	29.9 (17.9-41.1)	26.0 (18.6-26.0)	23.4 (17.4-40.3)	0.486	0.535
AST, range (U/L)	25.3 (19.4-34.8)	24.0 (19.1-30.6)	24.2 (19.1-33.3)	0.528	0.720
ALP, range (U/L)	75.7 (64.2-89.7)	74.7 (61.9-91.4)	73.5 (62.0-88.1)	0.829	0.574
GGT, range (U/L)	45.5 (26.3-65.5)	35.0 (21.0-58.5)	34.0 (21.0-61.3)	0.166	0.207
AFP, range (ng/mL)	11.0 (4.2-219.3)	6.9 (3.0-109.6)	6.9 (6.9-255.7)	0.163	0.333
CA19-9, range(U/mL)	13.4 (8.3-26.9)	13.8 (8.5-26.0)	13.6 (8.2-26.8)	0.967	0.841
PT, range (second)	13.4 (13.1-14.0)	13.3 (12.9-13.9)	13.2 (12.8-13.7)	0.327	0.137
PLT, range (10^9/L)	164.5 (130.0-213.0)	154.0 (123.5-204.0)	163.0 (129.0-216.8)	0.708	0.427
Transfusion, Yes/No	1/39 (2.5%/97.5%)	5/156 (3.1%/96.9%)	4/76 (5.0%/95.0%)	1.000	0.518
Diameter, range (cm)	3.5 (2.0-4.5)	2.8 (2.0-3.8)	3.1 (2.0-4.2)	0.035	0.714
Intact capsule, Yes/No	18/22 (45.0%/55.0%)	66/95 (41.0%/59.0%)	34/46 (42.5%/57.5%)	0.646	0.794
Differentiation, I/II+III/IV*	4/29/7 (10.0%/72.5%/17.5%)	23/119/19 (14.3%/73.9%/11.8%)	9/61/10 (11.2%/76.3%/12.5%)	0.542	0.519
Liver cirrhosis, Yes/No	28/12 (70.0%/30.0%)	96/65 (59.6%/40.4%)	49/31 (61.3%/38.8%)	0.227	0.346
BCLC Stage, 0/A	12/28 (30.0%/70.0%)	52/109 (32.3%/67.7%)	21/59(26.3%/73.8%)	0.780	0.665
Chinese Stage, Ia	40 (100.0.%)	161 (100.0.%)	80 (100.0.%)	–	–
TNM stage (AJCC, 8th), T1a/T1b	12/28 (30.0%/70.0%)	52/109 (32.3%/67.7%)	21/59(26.3%/73.8%)	0.780	0.665

“*” Classification of Edmondson-Steiner; TACE, transarterial chemoembolization; PSM, propensity score matching; TBIL, total bilirubin; ALB, albumin; ALT, alanine transaminase; AST, aspartate aminotransferase; ALP, alkaline phosphatase; GGT, γ-glutamyl transpeptidase; AFP, alpha fetal protein; CA19-9, Carbohydrate antigen19-9; PT, prothrombin time; PLT, platelets. P_1_ values, adjuvant TACE VS non-adjuvant TACE before a PSM; P_2_ values, adjuvant TACE VS non-adjuvant TACE after a PSM; BCLC stage, Barcelona Clinic Liver Cancer stage; TNM stage, tumor node metastasis staging system; AJCC, American Joint Committee on Cancer.

**Table 2:** Clinicopathological features of hepatocellular carcinoma patients with low risk of recurrence after liver resection (Eastern Hepatobiliary Surgery Hospital Cohorts).

Clinicopathological features	Adjuvant TACE (n=113)	Non-adjuvant TACE (n=175)	Non-adjuvant TACE after a PSM 1:1 (n=113)	*P_1_* values	*P_2_* values
Sex, female/male	15/98 (13.3%/86.7%)	28/147 (16.0%/84.0%)	17/96 (15.0%/85.0%)	0.526	0.703
Age, range (years)	52.0±9.3 (28.0-76.0)	53.0±11.1 (22.0-83.0)	53.4±11.1 (22.0-83.0)	0.409	0.299
Hepatitis, Yes/No	107/6 (94.7%/5.3%)	164/11 (93.7%/6.3%)	105/8 (92.9%/7.1%)	0.731	0.581
TBIL, (μmol/L)	13.4 (10.1-17.6)	14.3 (11.2-17.1)	13.3 (10.8-16.7)	0.561	0.143
ALB, range (g/L)	42.5±4.2 (29.4-53.4)	42.5±4.0 (31.4-51.7)	42.4±4.0 (31.4-51.5)	0.923	0.988
ALT, range (U/L)	35.4 (27.3-60.5)	33.1 (24.0-47.0)	35.8 (24.3-48.4)	0.060	0.201
AST, range (U/L)	31.8 (23.7-43.3)	29.0 (23.5-38.8)	29.0 (23.9-38.7)	0.175	0.221
ALP, range (U/L)	74.0 (61.5-89.5)	76.0 (61.0-89.0)	76.0 (61.0-86.5)	0.732	0.826
GGT, range (U/L)	47.0 (33.0-80.5)	41.0 (28.0-69.0)	41.0 (28.0-69.0)	0.117	0.137
AFP, range (ng/mL)	30.4 (5.8-262.6)	15.5 (4.8-324.3)	16.4 (4.8-388.0)	0.532	0.744
CA19-9, range(U/mL)	20.2 (11.9-32.6)	20.0 (10.4-32.3)	20.4 (10.5-32.9)	0.317	0.603
PT, range (second)	12.0 (11.6-13.1)	12.0 (11.5-12.7)	12 (11.5-12.5)	0.416	0.110
PLT, range (10^9/L)	143 (100.5-181.0)	133.0 (99.0-179.0)	139.0 (96.5-178.0)	0.501	0.450
Transfusion, Yes/No	8/105 (7.1%/92.9%)	28/147 (16.0%/84.0%)	8/105 (7.1%/92.9%)	0.025	1.000
Diameter, range (cm)	3.1 (2.5-4.0)	3.0 (2.1-4.0)	3.0 (2.5-4.0)	0.380	0.393
Intact capsule, Yes/No	60/53 (53.1%/46.9%)	76/99 (43.4%/56.6%)	68/45 (60.2%/39.8%)	0.563	0.283
Differentiation, I/II+III/IV*	11/71/31 (9.3%/62.8%/27.4%)	19/112/44 (10.9%/64.0%/25.1%)	14/64/35 (12.4%/56.6%/31.0%)	0.889	0.617
Liver cirrhosis, Yes/No	77/36 (68.1%/31.9%)	117/58 (66.9%/33.1%)	68/45 (60.2%/39.8%)	0.820	0.212
BCLC Stage, 0/A	11/102 (9.7%/90.3%)	36/139 (20.6%/79.4%)	8/105 (7.1%/92.9%)	0.015	0.472
Chinese Stage, Ia	113 (100.0.%)	175 (100.0.%)	113 (100.0.%)	-	-
TNM stage (AJCC, 8th), T1a/T1b	11/102 (9.7%/90.3%)	36/139 (20.6%/79.4%)	8/105 (7.1%/92.9%)	0.015	0.472

“*” Classification of Edmondson-Steiner; TACE, transarterial chemoembolization; PSM, Propensity Score Matching; TBIL, total bilirubin; ALB, albumin; ALT, alanine transaminase; AST, aspartate aminotransferase; ALP, alkaline phosphatase; GGT, γ-glutamyl transpeptidase; AFP, alpha fetal protein; CA19-9, Carbohydrate antigen19-9; PT, prothrombin time; PLT, platelets. P_1_ values, adjuvant TACE VS non-adjuvant TACE before a PSM; P_2_ values, adjuvant TACE VS non-adjuvant TACE after a PSM; BCLC stage, Barcelona Clinic Liver Cancer stage; TNM stage, tumor node metastasis staging system; AJCC, American Joint Committee on Cancer.

### Recurrence and OS in the SHCC cohorts and EHBH cohorts

The median follow-up duration of the SHCC cohort was 53.2 ± 2.3 months; the adjuvant TACE group was 53.4 ± 2.6 months and the non-adjuvant TACE groups was 51.1 ± 4.1 months. Intrahepatic recurrence occurred in 62 (30.8%, 62/201) patients, extrahepatic metastases occurred in 7 (3.5%, 7/201) patients, and death occurred in 19 (9.5%, 19/201) patients. The 1-, 3- and 5-year recurrence rates were 8.5%, 26.9% and 35.1%, respectively. The 1-, 3- and 5-year survival rates were 99.5%, 95.9% and 84.9%, respectively.

In the EHBH cohorts, the median follow-up was more than 60 months and the adjuvant TACE and non-adjuvant TACE groups were also more than 60 months. Intrahepatic recurrence occurred in 109 (37.8%, 109/288) patients, extrahepatic metastases occurred in 9 (3.1%, 9/288) patients, and death occurred in 25 (8.7%, 25/288) patients. The 1-, 3- and 5-year recurrence rates were 10.1%, 25.3% and 39.0%, respectively. The 1-, 3- and 5-year survival rates were 98.6%, 94.7% and 91.1%, respectively.

### Prognostic factors of recurrence and OS in SHCC cohorts

**Figure 1:**
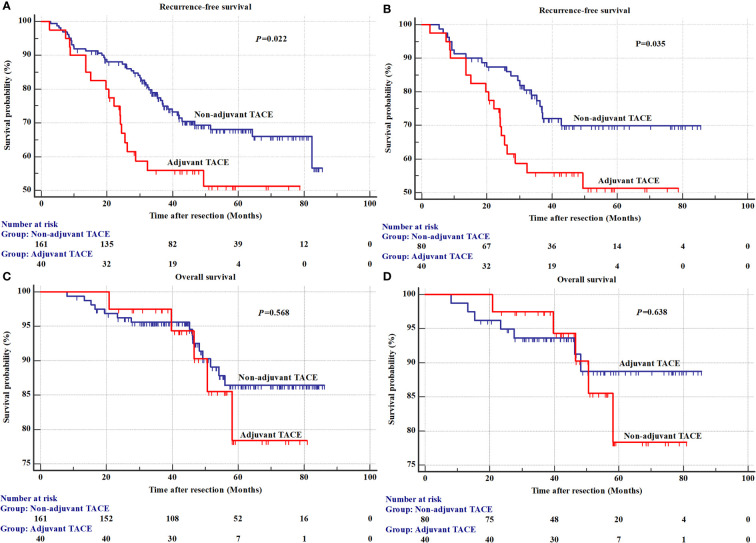
The recurrence and overall survival between adjuvant TACE and non-adjuvant TACE after hepatectomy in Shanghai Cancer Center cohorts. **(A)** Recurrence before a propensity score matching analysis; **(B)** Overall survival before a propensity score matching analysis; **(C)** Recurrence after a propensity score matching analysis; **(D)** Overall survival after a propensity score matching analysis. TACE, transarterial chemoembolization.

The results of univariate analysis for recurrence and OS in the SHCC cohorts before PSM are shown in **Supplementary Table 1** (**Figure 1**). The mean RFS was 51.0±4.8 months in the adjuvant TACE group, with 1-, 3-, and 5-year recurrence rates of 10.0%, 44.1% and 48.7%, respectively, and RFS was 66.0±2.4 months, with 1-, 3-, and 5-year recurrence rates of 8.1%, 22.6% and 31.9%, respectively, in the non-adjuvant TACE group. Kaplan–Meier curves showed that patients treated with adjuvant TACE had higher recurrence rates and shorter RFS after hepatectomy (**Figure 1A**, *P*=0.022). However, no significant difference was found in OS between adjuvant TACE and non-adjuvant TACE patients (**Figure 1C**, *P*=0.568). Cox proportional hazards multivariate analysis revealed that adjuvant TACE and serum ALP were independent prognostic factors for recurrence (**Table 3**). After PSM, the Kaplan–Meier curves still showed that patients with adjuvant TACE had higher recurrence rates and shorter RFS after hepatectomy. No significant difference existed in OS between these two groups (**Figure 1B**, *P*=0.035; **Figure 1D**, *P*=0.638). Adjuvant TACE was the only independent prognostic factor for recurrence, with a hazard ratio (HR) of 1.95 (**Table 3**, 95% confidence interval (CI): 1.04-3.66, *P*=0.038) by multivariate analysis (**Supplementary Table 2**; **Table 3**).

**Table 3:** Multivariate analysis of clinicopathological parameters associated with recurrence for hepatocellular carcinoma patients with low risk of recurrence after liver resection.

Clinicopathological parameters	HR	95% CI	*P* values
Before a PSM (SHCC Cohorts)			
Alkaline phosphatase, (U/L)	1.01	1.003-1.023	0.011
Adjuvant TACE, Yes/No	1.86	1.076-3.222	0.026
After a PSM (SHCC Cohorts)			
Adjuvant TACE, Yes/No	1.95	1.038-3.663	0.038
Before a PSM (EHBH Cohorts)			
Alkaline phosphatase, (U/L)	1.01	1.001-1.013	0.023
Adjuvant TACE, Yes/No	1.62	1.113-2.356	0.012
After a PSM (EHBH Cohorts)			
Adjuvant TACE, Yes/No	1.57	1.029-2.383	0.036

HR, hazard ratios; CI, confidence interval; PSM, propensity score matching; SHCC, Shanghai Caner Center; TACE, transarterial chemoembolization; EHBH, Eastern Hepatobiliary Surgery Hospital.

### Prognostic factors of recurrence and OS in EHBH cohorts

**Figure 2:**
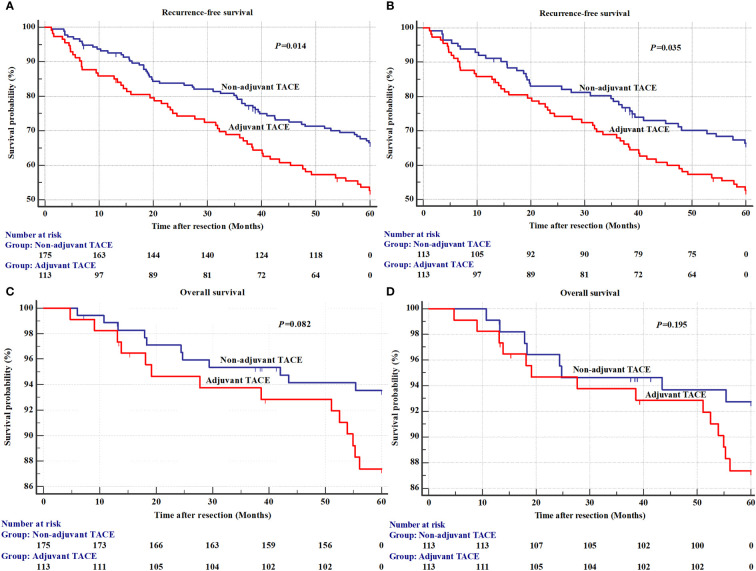
The recurrence and overall survival between adjuvant TACE and non-adjuvant TACE after hepatectomy in Eastern Hepatobiliary Surgery Hospital (EHBH) Cohorts. **(A)** Recurrence before a propensity score matching analysis; **(B)** Overall survival before a propensity score matching analysis; **(C)** Recurrence after a propensity score matching analysis; **(D)** Overall survival after a propensity score matching analysis. TACE, transarterial chemoembolization.

The results of univariate analysis for recurrence and OS in the EHBH cohorts before PSM are shown in **Supplementary Table 3**. The mean RFS was 43.6±2.0 months in the adjuvant TACE group, with 1-, 3-, and 5-year recurrence rates of 14.2%, 32.1% and 47.3%, respectively, and RFS was 49.3±1.4 months, with 1-, 3-, and 5-year recurrence rates of 7.4%, 22.1% and 33.5%, respectively, in the non-adjuvant TACE group. Kaplan–Meier curves also showed a significant difference in recurrence but not in OS between adjuvant TACE and non-adjuvant TACE patients after hepatectomy (**Figure 2A**, *P*=0.014; **Figure 2C**, *P*=0.082). Cox proportional hazards multivariate analysis also revealed that adjuvant TACE and serum ALP were both independent prognostic factors for recurrence (**Table 3**). Similarly, adjuvant TACE remained the only independent prognostic factor for recurrence, with an HR of 1.57 (**Table 3**, 95% CI: 1.03-2.38, *P*=0.036) by multivariate analysis after PSM (**Figures 2B, D**; **Table 3**; **Supplementary Table 4**).

## Discussion

Postoperative recurrence is the main obstacle to improving surgical efficacy in HCC (23). Increasing supporting evidence has confirmed that adjuvant TACE can benefit the high-risk recurrence population (4, 5, 12–18). However, it remains unclear whether adjuvant TACE can benefit patients with a low risk of recurrence after hepatectomy. In our study, PSM analysis revealed that patients receiving adjuvant TACE had significantly shorter RFS than those who did not, and that adjuvant TACE could not prolong the OS for patients with a low risk of recurrence after hepatectomy.

As early as 2004, Ren reported that in HCC patients with risk factors (tumors with a size greater than 5 cm, multiple nodules, and vascular invasion) for residual tumors after hepatectomy, adjuvant TACE could prolong survival but not in patients without risk factors for residual tumors (9). Since then, clinical studies have begun to pay attention to the relationship between adjuvant TACE and postoperative recurrence risk (13, 24–28). They also reported that adjuvant TACE can significantly reduce recurrence risk and prolong RFS and OS in HBV-related HCC patients with intermediate (a single tumor > 5 cm without MVI) or high risk of recurrence (single tumor with MVI or 2 or 3 tumors). However, patients with a low recurrence risk (single tumor ≤ 5 cm without MVI) were not included (13).

In addition, numerous meta-analyses have performed independent analyses of the risk factors for residual tumors (14, 16, 29–36). Among them, 5 studies performed subgroup analysis on the low risk of recurrence population (30–32, 34, 35). In 2014, Cheng reported that in HCC patients with a tumor size ≤ 5 cm, adjuvant TACE did not benefit disease-free survival (DFS) (30). Qi also found that in patients with small HCC (size ≤ 5 cm) or without vascular invasion, no significant differences existed in DFS and OS between the adjuvant TACE and adjuvant TACE groups (31). However, another study indicated that adjuvant TACE could benefit OS but not RFS in HCC patients with a tumor size < 5 cm (32). In addition, Huo also found that adjuvant TACE can significantly improve 1-year DFS and 5-year OS in HCC patients with a tumor size ≤ 5 cm and prolong 5-year DFS in patients without MVI but with OS (34). In contrast, Chen reported that in HCC patients with a tumor size ≤ 5 cm, a single tumor or MVI negativity, adjuvant TACE could not improve outcomes and could potentially promote recurrence after resection (35). The latest retrospective study also showed that adjuvant TACE might promote postoperative recurrence, especially for HCC patients without MVI, tumor size ≤ 5 cm and preoperative AFP < 400 ng/ml (37).

In our study, patients with a high risk of recurrence, such as those with multiple nodules, macrovascular invasion, microvascular invasion, satellites and larger tumor size (>5 cm), were excluded, and a total of 489 HCC patients (201 from SHCC and 288 from EHBH) were included. All patients had early-stage HCC (BCLC Stage 0 or A, Chinese Stage Ia and TNM Stage TIa or TIb; **Tables 1**, **2**). Comparatively, these patients have a lower risk of recurrence and longer long-term survival after hepatectomy. In the SHCC cohorts, 40 HCC patients (19.9%, 40/201) who received adjuvant TACE after hepatectomy had significantly shorter RFS than patients who did not receive adjuvant TACE (**Figure 1A**, *P*=0.022). However, no significant difference existed in OS (**Figure 1C**, *P*=0.568). The Cox proportional hazards multivariate analysis indicated that serum ALP and adjuvant TACE were both independent prognostic factors for recurrence (**Table 3**). After PSM analysis to balance the differences between the adjuvant TACE and non-adjuvant TACE groups, the Kaplan–Meier curves showed a significant difference in RFS, but not in OS, between adjuvant TACE and non-adjuvant TACE patients after hepatectomy (**Figure 1B**, *P*=0.035; **Figure 1D**, *P*=0.638). Multivariate analysis revealed that adjuvant TACE was the only independent prognostic factor for recurrence (**Table 3**). Similar results were also acquired in the EHBH cohorts (**Figure 2**; **Table 3**). The study also revealed that patients receiving TACE after hepatectomy had significantly shorter RFS before or after PSM, and adjuvant TACE was an independent prognostic factor for recurrence by multivariate analysis. It seems that in the low risk of recurrence population, adjuvant TACE could promote postoperative recurrence after hepatectomy but had no significant effect on OS. This outcome might be caused by liver function injury and immunological function damage induced by TACE (10, 11, 38). Recently, some models for predicting adjuvant TACE benefit in HCC patients have been proposed (39–42). They might constitute effective ways to select the population who can benefit from it.

There are several limitations of our study. First, it was a retrospective study with the inherent defects associated with such studies even after PSM, and a prospective study is required to validate the conclusions. Second, adjuvant TACE after resection usually required a comprehensive decision from the surgeon according to intraoperative and postoperative examinations. Because of selection bias, the recurrence risk of patients receiving adjuvant TACE may be higher than that of patients not receiving adjuvant TACE. Even if they received adjuvant TACE after resection, their outcomes may not be better than the outcomes of those who did not. Third, the cohorts come from two different institutions but were treated during different time periods (SHCC cohorts from March 2015 through September 2019; EHBH cohorts from December 2009 through June 2010). Treatment advances might have affected the prognoses of HCC patients and incurred recurrence and survivorship bias. Fourth, the mechanism for adjuvant TACE promoting postoperative recurrence need exploring.

## Conclusion

In summary, we focused on HCC patients with a low risk of recurrence reassessed whether adjuvant TACE could benefit prognosis in these populations and found that adjuvant TACE may not improve long-term survival and might promote postoperative recurrence in HCC patients with a low risk of recurrence after hepatectomy. More clinical trials with higher levels of evidence are needed and could help clinicians perform better interventions for these patients after curative resection.

## Data availability statement

The raw data supporting the conclusions of this article will be made available by the authors, without undue reservation.

## Ethics statement

This study was approved by the Clinical Research Ethics Committee of Eastern Hepatobiliary Surgery Hospital and Institutional Review Board and the Ethics Committee of Shanghai Cancer Center. The patients/participants provided their written informed consent to participate in this study.

## Author contributions

Study concept and design (L-HF, Y-YZ, J-MZ, W-MC, HD, and LW), acquisition of data (J-MZ, MW, W-QX, TZ, and A-RM), analysis and interpretation of data (L-HF, Y-YZ, J-MZ, W-MC, HD, and LW), drafting of the manuscript (L-HF, Y-YZ, and J-MZ), critical revision of the manuscript for important intellectual content (W-MC, HD, and LW), administrative, technical, or material support (MW, W-QX, TZ, and A-RM), and study supervision (W-MC, HD, and LW). All authors contributed to the article and approved the submitted version.
