# Supplementary material for: Adjuvant TACE may not improve recurrence-free or overall survival in HCC patients with low risk of recurrence after hepatectomy
Source: Front Oncol. 2023 May 24;13:1104492. doi: 10.3389/fonc.2023.1104492 (PMC10244569; doi:10.3389/fonc.2023.1104492)
Supplement: Supplementary file 1 [file DataSheet_1.pdf]

Title

Adjuvant TACE May Not Improve Recurrence-Free or Overall Survival in HCC Patients with Low Risk of Recurrence after Hepatectomy

Authors:

Long-Hai Feng[1, 2†], Yu-Yao Zhu[3†], Jia-Min Zhou[1, 2†], Miao Wang[1, 2], Wei-Qi Xu[1, 2], Ti Zhang[1, 2], An-Rong Mao[1, 2], Wen-Ming Cong[3*], Hui Dong[3*], Lu Wang[1, 2*]

Author Affiliations:

1. Department of Hepatic Surgery, Shanghai Cancer Center, Fudan University, Shanghai, China;

2. Department of Oncology, Shanghai Medical College, Fudan University, Shanghai, China;

3. Department of Pathology, Eastern Hepatobiliary Surgery Hospital, The Second Military Medical University, Shanghai, China;

†, These authors contributed equally to this work.

Corresponding author.

Lu Wang, Department of hepatic surgery, Shanghai Cancer Center, Fudan University, Shanghai 200032, P. R. China.

Email: wangluzl@fudan.edu.cn;

Telephone: 021-64175590

Hui Dong, Department of Pathology, Eastern Hepatobiliary Surgery Hospital, The Second Military Medical University, 225 Changhai Road, Shanghai, China.

Email: huidong@smmu.edu.cn

Wen-Ming Cong, Department of Pathology, Eastern Hepatobiliary Surgery Hospital, The Second Military Medical University, 225 Changhai Road, Shanghai, China.

Email: wmcong@outlook.com

Telephone: 021-81875191

Supplementary Materials - Index

Supplementary Figures and Tables

Supplementary Figure 1 *pag. 3*

Supplementary Figure 2 *pag. 4*

Supplementary Figure 3 *pag. 5*

Supplementary Table 1 *pag. 6*

Supplementary Table 2 *pag. 8*

Supplementary Table 3 *pag. 10*

Supplementary Table 4 *pag. 12*

Supplementary Figures and Tables

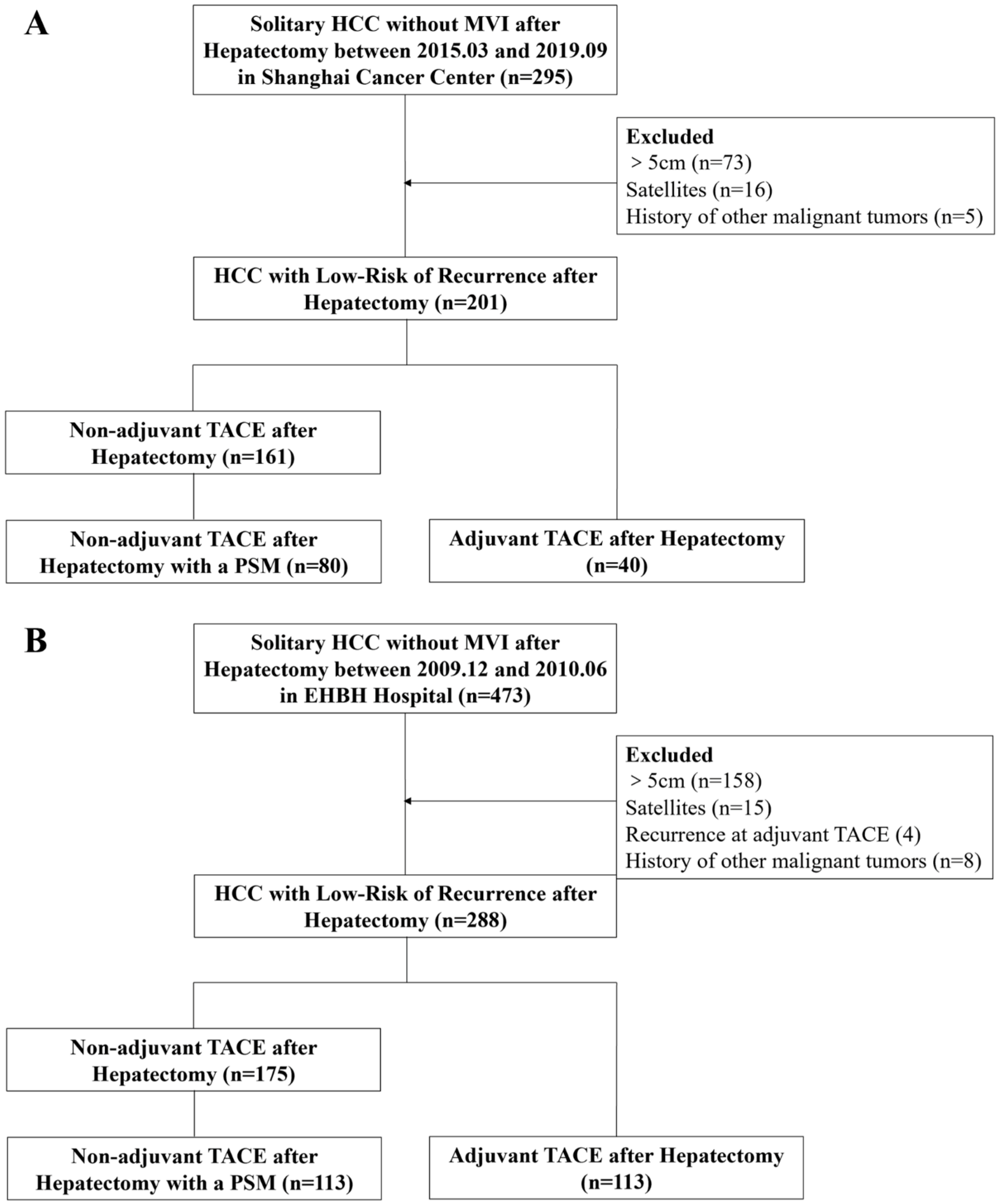

Supplementary Figure 1. The flow charts of our study. (A) Shanghai Cancer Center cohorts;(B). Eastern Hepatobiliary Surgery Hospital (EHBH) Cohorts. HCC, hepatocellular carcinoma; MVI, microvascular invasion. PSM, propensity score matching.

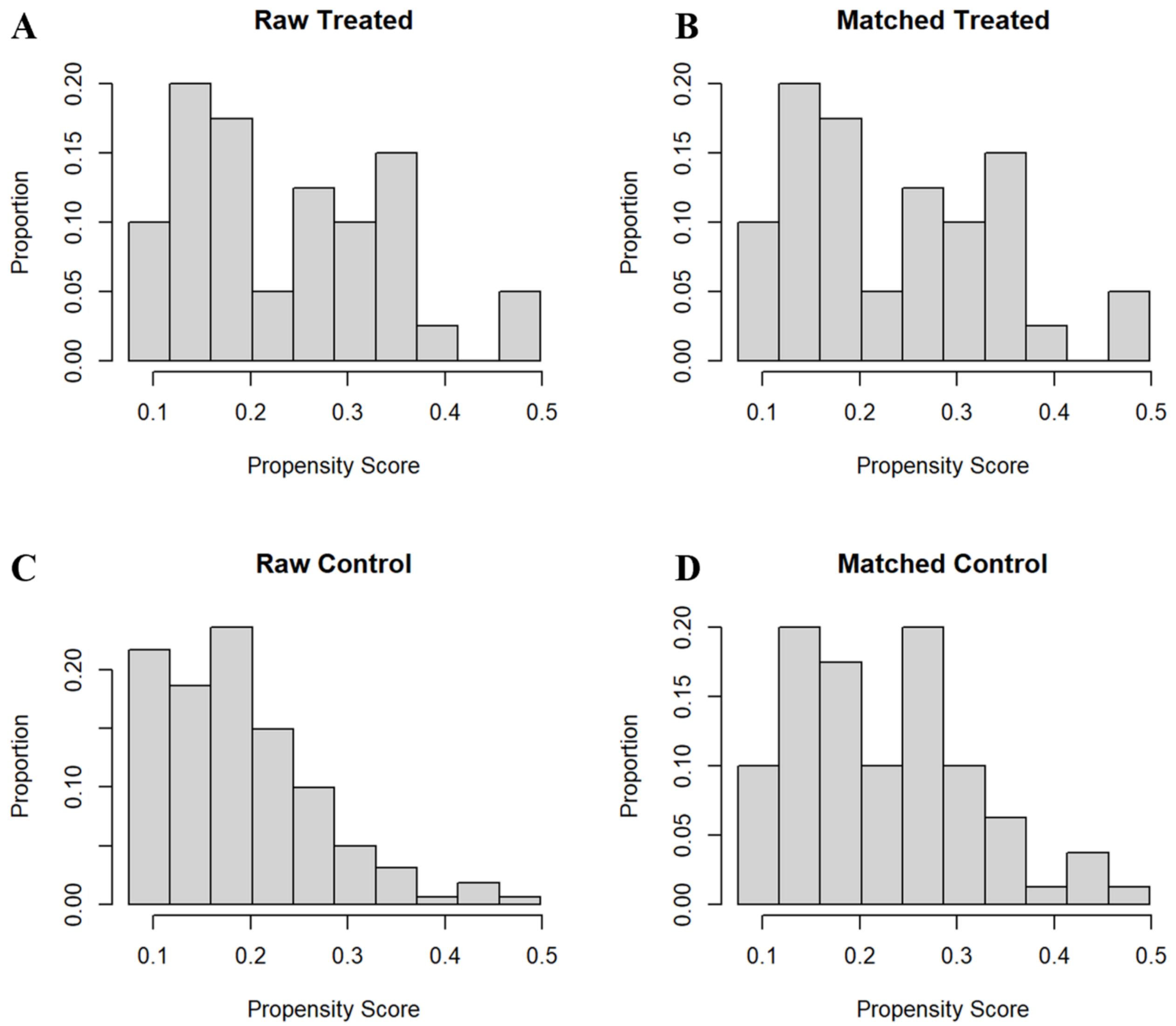

Supplementary Figure 2. Histograms of propensity scores before (A, C) and after (B, D) matching in Shanghai Cancer Center cohorts

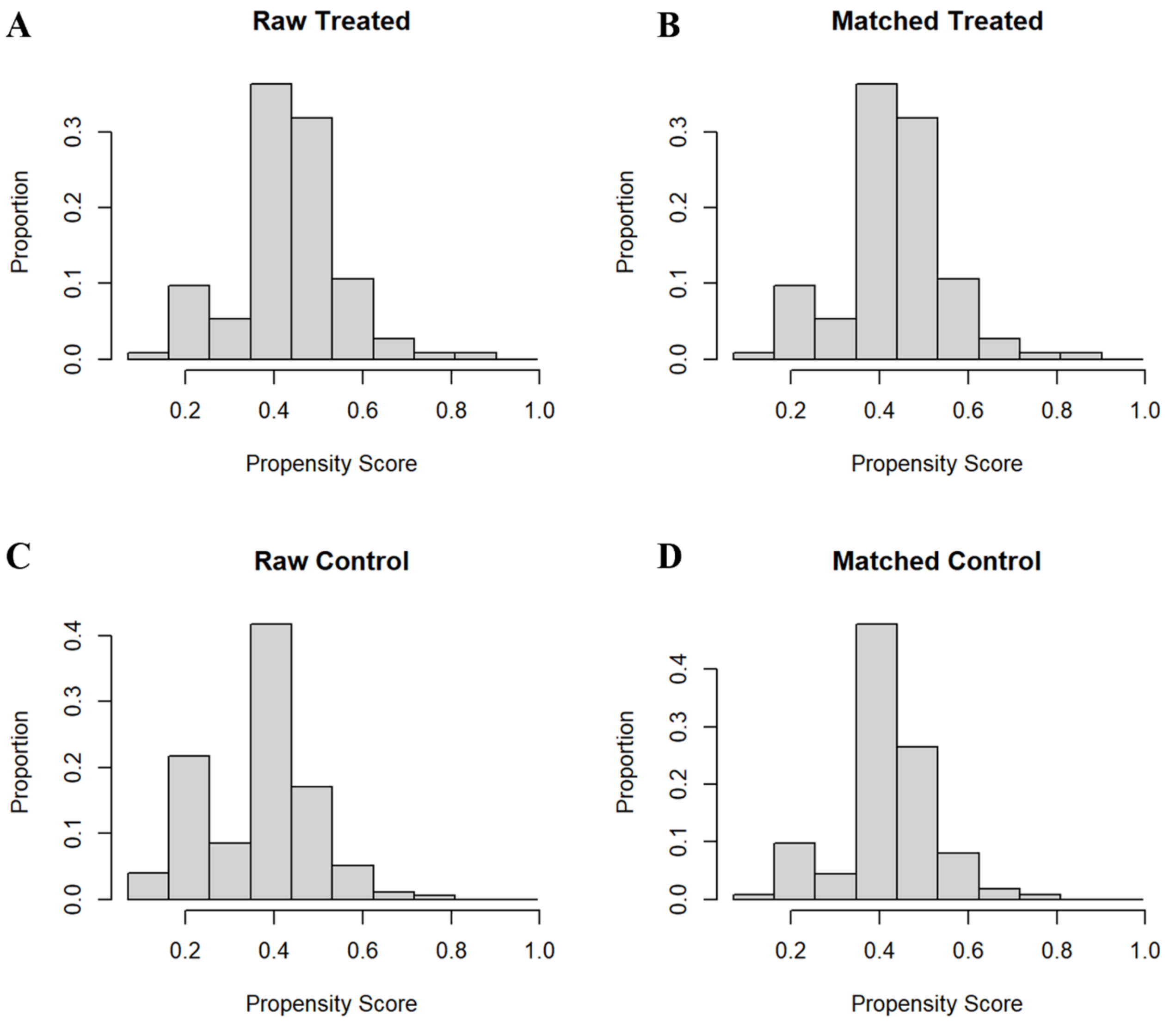

Supplementary Figure 3. Histograms of propensity scores before (A, C) and after (B, D) matching in Eastern Hepatobiliary Surgery Hospital Cohorts.

Supplementary Table 1. Univariate Analysis of Clinicopathological Parameters Associated with Recurrence and Overall Survival of Hepatocellular Carcinoma with Low-Risk of Recurrence after Liver Resection in the SHCC Cohorts before a PSM

Clinicopathological parameters	SHCC cohorts (n=201)	Recurrence			Overall survival		
		HR	95% CI	*P* values	HR	95% CI	*P* values
Sex, male/female	173/28 (86.1%/13.9%)	0.69	0.358-1.315	0.257	0.48	0.173-1.315	0.153
Age, range (years)	56.6±10.9 (31.0-84.0)	0.99	0.965-1.009	0.233	1.03	0.984-1.068	0.241
Hepatitis, Yes/No	155/46 (77.1%/22.9%)	1.19	0.631-2.222	0.598	0.59	0.227-1.547	0.285
TBIL, (μmol/L)	11.7 (9.1-16.0)	0.99	0.945-1.036	0.645	1.03	0.953-1.110	0.468
ALB, range (g/L)	44.0±3.6 (33.3-44.0)	1.00	0.931-1.068	0.941	1.00	0.886-1.133	0.977
ALT, range (U/L)	26.1 (18.4-38.4)	1.00	0.993-1.008	0.928	1.01	0.998-1.014	0.124
AST, range (U/L)	24.3 (19.2-30.6)	1.00	0.991-1.009	0.977	1.01	0.996-1.014	0.285
ALP, range (U/L)	74.9 (63.1-91.0)	1.01	1.003-1.024	**0.011**	1.02	0.997-1.035	0.091
GGT, range (U/L)	36.0 (23.0-59.5)	1.00	0.999-1.007	0.137	1.00	0.998-1.011	0.175
AFP, range (ng/mL)	7.3 (3.2-123.4)	1.00	1.000-1.000	0.148	1.00	1.00-1.00	0.056
CA19-9, range(U/mL)	13.7 (8.5-26.0)	1.01	0.993-1.017	0.442	1.03	1.015-1.047	**<0.001**
PT, range (second)	13.3 (13.0-13.9)	1.12	0.784-1.589	0.542	1.45	0.816-2.575	0.205
PLT, range (10^9/L)	155.0 (124.5-204.0)	1.00	0.995-1.004	0.766	1.00	0.993-1.008	0.882
Transfusion, Yes/No	6/195 (3.0%/97.0%)	0.97	0.236-3.939	0.964	0.05	0.000-4092.605	0.599
Adjuvant TACE, Yes/No	40/161 (19.9%/80.1%)	1.88	1.084-3.242	**0.024**	1.34	0.487-3.694	0.569
Diameter, range (cm)	2.9 (2.0-4.0)	1.01	0.817-1.239	0.952	1.47	1.014-2.134	**0.042**
Intact capsule, No/Yes	84/117 (41.8%/58.2%)	1.16	0.698-1.918	0.572	1.12	0.447-2.821	0.804
Differentiation, I/II+III/IV*	27/148/26 (13.4%/73.6%/12.9%)	0.80	0.495-1.288	0.798	1.33	0.575-3.080	0.504
Liver cirrhosis, Yes/No	124/77 (61.7%/38.3%)	1.40	0.831-2.365	0.205	1.50	0.576-3.904	0.406
BCLC Stage, 0/A	64/137 (31.8%/68.2%)	1.17	0.685-2.007	0.561	5.23	1.211-22.565	**0.027**

Chinese Stage, Ia	288 (100.0%)	-	-	-	-	-	-
TNM stage (AJCC, 8th), T1a/T1b	64/137 (31.8%/68.2%)	1.17	0.685-2.007	0.561	5.23	1.211-22.565	0.027

"*" Classification of Edmondson-Steiner; SHCC, Shanghai Caner Center; PSM, propensity score matching; HR, hazard ratios; CI, confidence interval; TBIL, total bilirubin; ALB, albumin; ALT, alanine transaminase; AST, aspartate aminotransferase; ALP, alkaline phosphatase; GGT, γ-glutamyl transpeptidase; AFP, alpha fetal protein; CA19-9, Carbohydrate antigen19-9; PT, prothrombin time; PLT, platelets. TACE, transarterial chemoembolization; BCLC stage, Barcelona Clinic Liver Cancer stage; TNM stage, tumor node metastasis staging system; AJCC, American Joint Committee on Cancer.

Supplementary Table 2. Univariate Analysis of Clinicopathological Parameters Associated with Recurrence and Overall Survival of Hepatocellular Carcinoma with Low-Risk of Recurrence after Liver Resection in the SHCC Cohorts after a PSM

Clinicopathological parameters	SHCC cohorts (n=120)	Recurrence			Overall survival		
		HR	95% CI	*P* values	HR	95% CI	*P* values
Sex, male/female	104/16 (86.7%/13.3%)	0.68	0.302-1.550	0.363	0.83	0.181-3.815	0.811
Age, range (years)	56.6±11.2 (31.0-84.0)	0.98	0.957-1.011	0.243	1.03	0.977-1.090	0.259
Hepatitis, Yes/No	89/31 (74.2%/25.8%)	1.94	0.812-4.628	0.136	0.83	0.222-3.067	0.773
TBIL, (μmol/L)	11.6 (8.8-15.2)	0.98	0.923-1.040	0.504	1.01	0.913-1.118	0.837
ALB, range (g/L)	43.6±3.6 (33.3-52.2)	1.05	0.961-1.146	0.282	1.02	0.874-1.188	0.808
ALT, range (U/L)	25.3 (17.6-40.3)	1.00	0.993-1.008	0.858	1.01	0.997-1.015	0.196
AST, range (U/L)	24.7 (19.3-33.3)	1.00	0.988-1.010	0.837	1.01	0.995-1.015	0.341
ALP, range (U/L)	73.9 (63.5-89.4)	1.01	0.999-1.026	0.065	1.01	0.989-1.039	0.279
GGT, range (U/L)	37.5 (23.0-62.5)	1.00	0.996-1.007	0.649	1.00	0.995-1.012	0.457
AFP, range (ng/mL)	8.8 (3.0-226.7)	1.00	1.000-1.000	0.141	1.00	1.000-1.001	0.053
CA19-9, range(U/mL)	13.4 (8.2-26.8)	1.00	0.985-1.016	0.951	1.03	1.009-1.049	**0.004**
PT, range (second)	13.3 (12.9-13.8)	1.32	0.844-2.072	0.223	1.39	0.620-3.02	0.427
PLT, range (10^9/L)	163.0 (129.8-216.8)	1.00	0.996-1.007	0.666	1.00	0.992-1.011	0.797
Transfusion, Yes/No	5/115 (4.2%/95.8%)	0.51	0.069-3.684	0.501	0.05	0.000-18857.294	0.642
Adjuvant TACE, Yes/No	40/80 (33.3%/66.7%)	1.95	1.038-3.663	**0.038**	1.32	0.417-4.151	0.639
Diameter, range (cm)	3.4 (2.0-4.5)	0.97	0.746-1.266	0.833	1.29	0.795-2.092	0.303
Intact capsule, No/Yes	52/68 (43.3%/56.7%)	1.09	0.572-2.051	0.806	1.02	0.322-3.205	0.978
Differentiation, I/II+III/IV*	13/90/17 (10.8%/75.0%/14.2%)	0.82	0.442-1.516	0.525	1.07	0.356-3.229	0.902
Liver cirrhosis, Yes/No	77/43 (64.2%/35.8%)	2.07	0.981-4.357	0.056	1.02	0.307-3.399	0.973
BCLC Stage, 0/A	33/87 (27.5%/72.5%)	0.87	0.439-1.712	0.681	2.23	0.487-10.200	0.302

Chinese Stage, Ia	120 (100.0%)	-	-	-	-	-	-
TNM stage (AJCC, 8th), T1a/T1b	33/87 (27.5%/72.5%)	0.87	0.439-1.712	0.681	2.23	0.487-10.200	0.302

"*" Classification of Edmondson-Steiner; SHCC, Shanghai Caner Center; PSM, propensity score matching; HR, hazard ratios; CI, confidence interval; TBIL, total bilirubin; ALB, albumin; ALT, alanine transaminase; AST, aspartate aminotransferase; ALP, alkaline phosphatase; GGT, γ-glutamyl transpeptidase; AFP, alpha fetal protein; CA19-9, Carbohydrate antigen19-9; PT, prothrombin time; PLT, platelets. TACE, postoperative adjuvant transarterial chemoembolization; BCLC stage, Barcelona Clinic Liver Cancer stage; TNM stage, tumor node metastasis staging system; AJCC, American Joint Committee on Cancer.

Supplementary Table 3. Univariate Analysis of Clinicopathological Parameters Associated with Recurrence and Overall Survival of Hepatocellular Carcinoma with Low-Risk of Recurrence after Liver Resection in the EHBH Cohorts before a PSM

Clinicopathological parameters	EHBH cohorts (n=288)	Recurrence			Overall survival		
		HR	95% CI	*P* values	HR	95% CI	*P* values
Sex, male/female	245/43 (85.1%/14.9%)	1.48	0.812-2.694	0.200	1.28	0.382-4.269	0.691
Age, range (years)	52.6±10.4 (22.0-83.0)	1.00	0.985-1.021	0.768	0.78	0.995-1.033	0.783
Hepatitis, Yes/No	271/17 (94.1%/5.9%)	1.56	0.636-3.825	0.332	22.13	0.017-29606.889	0.399
TBIL, (μmol/L)	13.9 (10.9-17.2)	0.99	0.968-1.015	0.458	0.99	0.935-1.045	0.681
ALB, range (g/L)	42.5±4.1 (29.4-53.4)	0.97	0.931-1.017	0.973	0.97	0.879-1.060	0.455
ALT, range (U/L)	34.3 (23.6-39.8)	1.00	0.995-1.003	0.506	0.99	0.973-1.006	0.212
AST, range (U/L)	30.1 (23.6-39.8)	1.00	0.995-1.004	0.761	0.99	0.965-1.010	0.272
ALP, range (U/L)	75.0 (61.0-89.0)	1.01	1.001-1.013	**0.030**	1.01	0.991-1.019	0.484
GGT, range (U/L)	44.0 (29.0-67.8)	1.00	1.000-1.004	0.053	1.00	0.996-1.006	0.735
AFP, range (ng/mL)	18.7 (5.4-309.4)	1.00	0.999-1.000	0.760	1.00	0.998-1.000	0.131
CA19-9, range(U/mL)	20.0 (10.8-32.2)	1.00	0.991-1.010	0.999	0.99	0.971-1.015	0.521
PT, range (second)	12.0 (11.5-12.9)	1.14	0.983-1.316	0.083	1.06	0.763-1.478	0.722
PLT, range (10^9/L)	140.0 (99.3-180.0)	1.00	0.997-1.003	0.946	1.00	0.997-1.009	0.257
Transfusion, Yes/No	36/252 (12.5%/87.5%)	1.03	0.585-1.796	0.931	0.29	0.039-2.156	0.227
Adjuvant TACE, Yes/No	113/175 (39.2%/60.8%)	1.59	1.094-2.311	**0.015**	1.98	0.902-4.378	0.088
Diameter, range (cm)	3.0 (2.3-4.0)	1.07	0.894-1.287	0.451	1.12	0.763-1.635	0.569
Intact capsule, No/Yes	129/159 (44.8%/55.2%)	0.96	0.661-1.404	0.846	2.25	0.993-5.085	0.052
Differentiation, I/II+III/IV*	30/183/75 (10.4%/63.5%/26.0%)	1.71	0.780-1.471	0.671	0.89	0.447-1.695	0.683
Liver cirrhosis, Yes/No	194/74 (67.4%/32.6%)	1.22	0.810-1.846	0.337	1.22	0.509-2.916	0.658

BCLC Stage, 0/A	47/241 (16.3%/83.7%)	1.08	0.650-1.786	0.772	1.05	0.361-3.060	0.928
Chinese Stage, Ia	288 (100.0%)	-	-	-		-	-
TNM stage (AJCC, 8th), T1a/T1b	47/241 (16.3%/83.7%)	1.08	0.650-1.786	0.772	1.05	0.361-3.060	0.928

"*" Classification of Edmondson-Steiner; EHBH, Eastern Hepatobiliary Surgery Hospital; PSM, propensity score matching; HR, hazard ratios; CI, confidence interval; TBIL, total bilirubin; ALB, albumin; ALT, alanine transaminase; AST, aspartate aminotransferase; ALP, alkaline phosphatase; GGT, γ-glutamyl transpeptidase; AFP, alpha fetal protein; CA19-9, Carbohydrate antigen19-9; PT, prothrombin time; PLT, platelets. TACE, transarterial chemoembolization; BCLC stage, Barcelona Clinic Liver Cancer stage; TNM stage, tumor node metastasis staging system, AJCC, American Joint Committee on Cancer.

Supplementary Table 4. Univariate Analysis of Clinicopathological Parameters Associated with Recurrence and Overall Survival of Hepatocellular Carcinoma with Low-Risk of Recurrence after Liver Resection in the EHBH Cohorts after a PSM

Clinicopathological parameters	EHBH cohorts (n=226)	Recurrence			Overall survival		
		HR	95% CI	*P* values	HR	95% CI	*P* values
Sex, male/female	194/32 (85.8%/14.2%)	1.15	0.611-2.159	0.667	1.02	0.303-3.462	0.969
Age, range (years)	52.7±10.2 (22.0-83.0)	1.01	0.992-1.032	0.253	1.00	0.963-1.045	0.878
Hepatitis, Yes/No	212/14 (93.8%/6.2%)	1.31	0.533-3.236	0.554	22.24	0.012-40470.098	0.418
TBIL, (μmol/L)	13.4 (10.6-17.2)	1.00	0.972-1.022	0.811	1.00	0.942-1.051	0.857
ALB, range (g/L)	42.5±4.1 (29.4-53.4)	0.97	0.926-1.021	0.262	0.96	0.867-1.056	0.381
ALT, range (U/L)	35.6 (25.6-51.6)	1.00	0.994-1.003	0.526	0.99	0.970-1.007	0.214
AST, range (U/L)	30.6 (23.7-40.0)	1.00	0.994-1.005	0.860	0.99	0.964-1.012	0.311
ALP, range (U/L)	74.0 (61.0-87.3)	1.01	1.000-1.017	0.056	1.01	0.987-1.023	0.623
GGT, range (U/L)	45.0 (29.0-72.0)	1.00	1.001-1.005	**0.007**	1.00	0.997-1.007	0.520
AFP, range (ng/mL)	20.6 (5.4-356.0)	1.00	1.000-1.000	0.951	1.00	0.998-1.000	0.157
CA19-9, range(U/mL)	20.3 (11.3-32.8)	1.00	0.989-1.010	0.923	0.99	0.965-1.013	0.375
PT, range (second)	12.0 (11.5-12.7)	1.17	1.000-1.374	**0.050**	1.12	0.784-1.562	0.564
PLT, range (10^9/L)	140.5 (97.8-181.0)	1.00	0.995-1.002	0.472	1.00	0.998-1.011	0.216
Transfusion, Yes/No	16/210 (7.1%/92.9%)	1.45	0.701-3.000	0.316	0.63	0.084-4.658	0.627
Adjuvant TACE, Yes/No	113/113 (50.0%/50.0%)	1.57	1.029-2.383	**0.036**	1.77	0.739-4.201	0.201
Diameter, range (cm)	3.1 (2.5-4.0)	1.19	0.957-1.488	0.117	1.14	0.732-1.768	0.565
Intact capsule, No/Yes	129/159 (44.8%/55.2%)	0.88	0.578-1.344	0.557	1.96	0.837-4.584	0.121

Differentiation, I/II+III/IV*	25/135/66 (11.1%/59.7%/29.2%)	1.11	0.798-1.556	0.525	0.97	0.489-1.918	0.927
Liver cirrhosis, Yes/No	145/81 (64.2%/35.8%)	1.29	0.828-2.021	0.259	1.45	0.567-3.705	0.438
BCLC Stage, 0/A	19/207 (8.4%/91.6%)	1.46	0.635-3.332	0.375	0.94	0.219-4.003	0.929
Chinese Stage, Ia	233 (100.0%)	-	-	-	-	-	-
TNM stage (8th), T1a/T1b	19/207 (8.4%/91.6%)	1.46	0.635-3.332	0.375	0.94	0.219-4.003	0.929

“*” Classification of Edmondson-Steiner; EHBH, Eastern Hepatobiliary Surgery Hospital; PSM, propensity score matching; HR, hazard ratios; CI, confidence interval; TBIL, total bilirubin; ALB, albumin; ALT, alanine transaminase; AST, aspartate aminotransferase; ALP, alkaline phosphatase; GGT, γ-glutamyl transpeptidase; AFP, alpha fetal protein; CA19-9, Carbohydrate antigen19-9; PT, prothrombin time; PLT, platelets. TACE, transarterial chemoembolization; BCLC stage, Barcelona Clinic Liver Cancer stage; TNM stage, tumor node metastasis staging system; AJCC, American Joint Committee on Cancer.
